# Hierarchical nanoporosity enhanced reversible capacity of bicontinuous nanoporous metal based Li-O_2_ battery

**DOI:** 10.1038/srep33466

**Published:** 2016-09-19

**Authors:** Xianwei Guo, Jiuhui Han, Pan Liu, Luyang Chen, Yoshikazu Ito, Zelang Jian, Tienan Jin, Akihiko Hirata, Fujun Li, Takeshi Fujita, Naoki Asao, Haoshen Zhou, Mingwei Chen

**Affiliations:** 1 WPI Advanced Institute for Materials Research, Tohoku University, Sendai 980-8577, Japan; 2 CREST, Japan Science and Technology Agency (JST), Saitama 332-0012, Japan; 3 Energy Technology Research Institute, National Institute of Advanced Industrial Science and Technology (AIST), Umezono 1-1-1, Tsukuba, 305-8568, Japan; 4 State Key Laboratory of Metal Matrix Composites, School of Materials Science and Engineering, Shanghai Jiao Tong University, Shanghai 200030, PR China

## Abstract

High-energy-density rechargeable Li-O_2_ batteries are one of few candidates that can meet the demands of electric drive vehicles and other high-energy applications because of the ultra-high theoretical specific energy. However, the practical realization of the high rechargeable capacity is usually limited by the conflicted requirements for porous cathodes in high porosity to store the solid reaction products Li_2_O_2_ and large accessible surface area for easy formation and decomposition of Li_2_O_2_. Here we designed a hierarchical and bicontinuous nanoporous structure by introducing secondary nanopores into the ligaments of coarsened nanoporous gold by two-step dealloying. The hierarchical and bicontinuous nanoporous gold cathode provides high porosity, large accessible surface area and sufficient mass transport path for high capacity and long cycling lifetime of Li-O_2_ batteries.

The working principle of the non-aqueous Li-O_2_ battery is based on an electrochemical reaction: 2Li^+^ +2e^−^ +O_2_↔Li_2_O_2_ (E_o_ = 2.96 V *vs* Li^+^/Li) with the solid Li_2_O_2_ formation and decomposition at cathodes^1^,^2^,^3^,^4^,^5^,^6^,^7^,^8^,^9^,^10^,^11^. Based on the reaction, the theoretical specific energy of Li-O_2_ battery is estimated to be ~3600 Wh/kg. However, the realization of a large rechargeable capacity of Li-O_2_ battery is facing many challenges. Since three phases (gas, liquid and solid) are involved into the electrode reactions during discharging and charging, the cathodes are required to be porous for fast transport of oxygen and electrolytes and for accommodation of the solid reaction products. Various conductive porous materials, such as carbon^12^,^13^,^14^,^15^,^16^,^17^,^18^,^19^,^20^,^21^,^22^,^23^,^24^,^25^,^26^,^27^,^28^,^29^,^30^,^31^,^32^,^33^, carbides^34^,^35^,^36^ and metals/composites^37^,^38^,^39^,^40^,^41^,^42^,^43^,^44^,^45^,^46^,^47^,^48^ have been widely explored as cathodes for Li-O_2_ batteries. Particularly, nanoporous gold (NPG), fabricated by dealloying, shows high kinetic efficiency for the Li_2_O_2_ formation and decomposition at low overpotentials. However, the attainable capacity from the NPG cathodes is very low (~300 mAh/g) because of the high mass density of gold electrode and the limited porosity (~50%) of NPG to host the discharge products^47^. In general, higher porosity with the low mass density of the porous cathodes can host more Li_2_O_2_ for higher capacity and, meanwhile, a larger accessible surface area promotes the reaction kinetics of Li_2_O_2_ formation and decomposition during discharging and charging. However, it is usually intolerable to realize both high porosity and large surface area simultaneously in porous materials because larger surface areas need a smaller pore size while higher porosity requires larger pores. Although the NPG cahode may not be an ideal choice for practical application of Li-O_2_ battery because of the usage of gold, it is an ideal system to study the solution of the conflict requirements because of the tunable nanopores in an controllable manner. Therefore, we developed a hierarchical nanoporous gold (*h*-NPG) cathode with a high effective surface area for Li_2_O_2_ formation/decomposition and large porosity for the storage of the solid products. The *h*-NPG based Li-O_2_ battery shows a highly enhanced reversible capacity of ~1500 mAh/g with over 140 cycles at low discharge/charge overpotentials.

## Results

**Figure 1:**
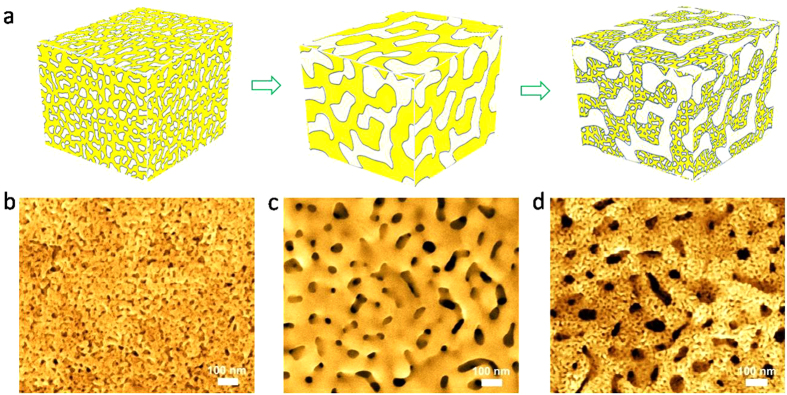
(**a)** Schematic diagram of the fabrication process of three-dimensional hierarchical NPG. SEM images of (**b**) as-prepared nanoporous AuAg alloy with an average nanopore size of ~20 nm; and (**c**) coarsened nanoporous AuAg alloy with a coarsened pore size of ~80–100 nm. (**d**) SEM image of the hierarchical nanoporous gold with ~5–20 nm small pores on the ~80–100 nm ligaments.

The *h*-NPG electrodes were prepared by a dealloying method (Fig. 1a)^49^,^50^,^51^,^52^. The constitute Ag in a Au_15_Ag_85_ (at%) alloy was partially leached away in an acid solution to form a nanoporous Au-Ag alloy (np-AuAg) with an average pore size of ~20 nm and residual Ag of ~70 at% (Figs 1b and S1). Subsequently, the np-AuAg was annealed in air and a coarsened nanoporous structure with pore and ligament sizes of ~80–100 nm was achieved (Fig. 1c). The residual Ag in the coarsened porous alloy was further selectively removed by second-step dealloying, leading to the formation of *h*-NPG with ~5–20 nm pores on ~80–100 nm ligaments (Fig. 1d). The distributions of pore sizes in the NPG samples with different morphology were carefully measured and plotted in Fig. S2. The *h-*NPG has a large porosity of ~80–82 *vol.*% and a large effective surface area of ~82.9 m^2^/g (Fig. S3). In contrast, the as-prepared and coarsened np-AuAg samples with ~50 *vol.*% porosity have the effective surface areas of ~59.8 and 27.4 m^2^/g, respectively (Table S1). Apparently, the hierarchical nanoporous configuration gives rise to both large effective surface area and high porosity, which cannot be achieved from conventional monolithic porous structure.

**Figure 2:**
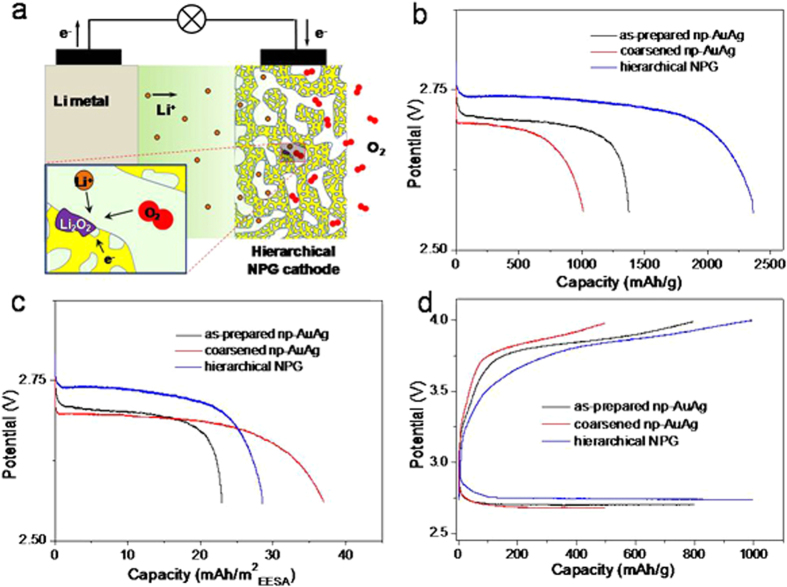
(**a**) Schematic diagram of the hierarchical NPG based Li-O_2_ battery. (**b**) The maximum capacities based on the mass of the nanoporous electrodes of the as-prepared np-AuAg, coarsened np-AuAg and hierarchical NPG. (**c**) The maximum capacities normalized by the electrochemical effective surface areas of the three NPG cathodes. The measurements were conducted in 1 M LiClO_4_ in DMSO at the current density of 0.5 A/g. The maximum capacities were determined at the cut-off voltage of 2.55 V. (**d**) The galvanostatic discharge/charge curves of the three batteries at the current density of 0.5 A/g with the terminal charge potential of 4.0 V.

The free-standing and bicontinuous NPG samples were directly used as the cathodes of Li-O_2_ batteries. Figure 2a shows the schematic configuration of the testing battery, in which *h*-NPG was utilized as the cathode and the propylene carbonate pretreated Li metal as the anode in a 1M LiClO_4_ dimethyl sulfoxide (DMSO) solution^47^,^48^. For comparison, the as-prepared and coarsened np-AuAg cathodes were also tested using the same setup. The three electrodes show the large difference in the maximum discharge capacity. The *h*-NPG has the highest discharge capacity of ~2400 mAh/g while the as-prepared and coarsened np-AuAg cathodes are only about 1400 and 1100 mAh/g, respectively (Figs 2b and S4a). All the capacities and current densities in this study are calculated by the weight of the cathodes. Because of the free-standing nature, the NPG cathodes do not have a current collector. Although the as-prepared and coarsened np-AuAg samples have a nearly identical porosity of ~50 *vol*%, the as-prepared sample with a larger specific surface area and a smaller pore size has higher capacity. This suggests that large effective surface areas provide more active sites for the formation of solid Li_2_O_2_, which is consistent with the conventional wisdom that the capacity is closely related to the effective surface of cathodes^9^. Interestingly, after normalizing the maximum discharge capacities using the electrochemically effective surface areas (EESA), the capacities noticeably depend on the porous morphology of the cathodes (Fig. 2c) while the dependence on the geometric surface area of NPG is weak (Fig. S4b). The coarsened np-AuAg has the largest normalized capacity, followed by the *h*-NPG and as-prepared np-AuAg (Fig. 2c). Apparently, the discharge capacity is not solely determined by the electrode surface areas, the geometric morphology of porous structure also plays a critical role in the maximum capacity. Although small pores with a high surface area are important to maximize active sites for oxygen reduction reaction (ORR) and hence a large discharge capacity, the ORR during discharging involves heavy mass transport and the solid reaction products of Li_2_O_2_ may block pore channels to prevent the effective utilization of the surface area in nanoporous structure. For coarsened porous structure, the large pore channels cannot be easily clogged by the solid reaction products and thus the effective surface area can be well exploited for ORR. However, the coarsened porous structure does not have a large surface area for a high capacity. Therefore, the battery performance of NPG cathodes is closely related to both morphology and accessible surface area of porous electrodes, which underlines the importance of the hierarchical nanoporous structure for the cathode design that can utilize concurrently large pore channels for operative mass transport, and small pores for a large effective surface area.

The maximum discharge capacity apparently correlates with the reversible capacity of NPG cathodes. The fully reversible capacity of the *h*-NPG cathode can reach ~1000 mAh/g at the cut-off charging potential of 4.0 V while the coarsened and as-prepared np-AuAg cathodes can only be ~500 and ~800 mAh/g, respectively (Fig. 2d). Interestingly, both charge and discharge overpotentials of the NPG cathodes show the noticeable dependence on the morphology of the nanoporous cathodes. The charge potential (Table S2) of the *h*-NPG is ~0.14 V and 0.07 V lower than that of the coarsened and as-prepared np-AuAg, respectively. This tendency could be explained by the fact that a smaller ligament/pore size yields a larger effective surface area and high density of geometrically required surface defects for enhanced oxidation reaction of Li_2_O_2_^53^,^54^. The discharge overpotential of the *h*-NPG is also lower than those of the coarsened and as-prepared np-AuAg as shown in Fig. 2d and Table S2. It has been verified that NPG is active for ORR^54^, and, particularly, the small nanopore channels of nanoporous metals can effectively enhance ORR kinetics by trapping oxygen at a low overpotential for multiple molecule/catalyst contacts^55^. Since the *h*-NPG contains much lower residual silver (~10 at%) in comparison with the as-prepared and coarsened np-AuAg (~70 at%), the chemical composition difference may play a role in the electrode reactions kinetics during discharging and charging. In order to investigate the possible chemical effect, we developed as-prepared and coarsened NPG with the same composition as the *h*-NPG with similar pore sizes as the as-prepared and coarsened np-AuAg (Fig. S5a–d). As shown in Fig. S5e, the battery performances of the NPG cathodes with the nearly identical chemical composition but different porous structures are analogous to Fig. 2d, suggesting that the cathodic performances of the nanoporous samples are primarily controlled by porous morphology. The insignificant effect of the residual silver in the discharge/charge reactions may be related to the fact that the internal surfaces of the nanoporous cathodes are passivated by pure gold by dealloying while the residual Ag only resides in the interior of gold ligaments and does not participate into the cathodic reactions^56^.

**Figure 3:**
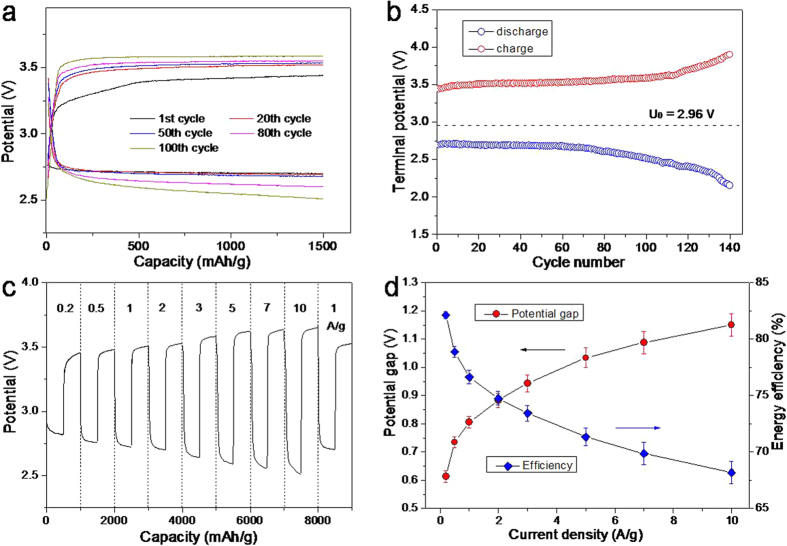
(**a**) The galvanostatic discharge/charge curves of the hierarchical NPG cathode at the current density of 2.0 A/g with the cut-off capacity of 1500 mAh/g. The electrolyte is 1 M LiClO_4_ in DMSO with 50 mM TTF. (**b**) The terminal potentials versus the cycle number of the hierarchical NPG based Li-O_2_ battery at the cut-off capacity of 1500 mAh/g. (**c**) The rate dependence of the Li-O_2_ battery with the cut-off capacity of 500 mAh/g and the current densities ranging from 0.2 A/g to 10.0 A/g. (**d**) The corresponding potential gap and energy efficiency versus current density. The potential gap is the sum of the discharge overpotential (η_dis_) and charge overpotential (η_cha_) at each testing current density. Here η_dis_ and η_cha_ are calculated by the terminal potentials.

Although the *h*-NPG cathode with an optimal hierarchical nanoporosity shows a high reversible capacity at low charge/discharge overpotentials, it does not have a good cycling retention and often fails with tens of cycles. This is probably limited by the slow kinetics of dissolving Li_2_O_2_ from deep nanopore channels. Following the work that the redox mediator, tetrathiafulvalene (TTF), can significantly enhance the charging kinetics of NPG cathodes^48^, we modified the 1M LiClO_4_/DMSO electrolyte by adding TTF and kept the Li metal plate as the anode. With this modified electrolyte, the galvanostatic charge curves of the three nanoporous cathodes become obviously flat with lower charge overpotentials (Fig. S6a), compared to those tested in the electrolyte without TTF (Fig. 2d). In contrast, the discharge potentials of the three electrodes do not show noticeably changes (Table S2), indicating that TTF mainly benefits the oxidation kinetics of Li_2_O_2_ during charging. It is worth noting that the discharge/charge overpotentials of the nanoporous cathodes still show the obvious porous morphology dependence after the electrolyte is modified by the redox mediator (Fig. S6a and Table S2). The *h*-NPG electrode keeps the lowest charge/discharge overpotentials, followed by the as-prepared and coarsened np-AuAg. Furthermore, the effect of TTF has been investigated by *cyclic voltammetry* (CV) of the electrode reactions with fixed scan potential window (Figs S6–S7)^22^ and *ex-situ* SEM after the battery was fully discharged and recharged for the first cycle (Fig. S8). Both of these characterizations have confirmed that the TTF enhanced Li_2_O_2_ oxidation takes place at the potentials above ~3.4–3.55 V, which contributes to the majority of the charge capacity achieved by the *h*-NPG based Li-O_2_ battery as shown in Fig. 3a.

With the redox mediator, the reversible capacity and cyclic retention of the *h-*NPG cathodes are dramatically improved. Although the fully reversible capacity cannot be obtained for long cycles, the *h*-NPG shows an excellent cycling retention at a high capacity of 1500 mAh/g for over 140 cycles at a high current density of 2.0 A/g (Fig. 3a). The discharge potential of the Li-O_2_ battery keeps the near constant of ~2.70 V for the initial 50 cycles and then gradually decreases to ~2.50 V after 100 cycles (Fig. 3b). The charge potential of the Li-O_2_ battery also shows a lower value of ~3.5 V for first 50 cycles and preserves below ~3.6 V even at 100^th^ cycle, which is much lower than those of other cathodes reported in the literature^3^. Furthermore, the *h*-NPG based batteries show the good cycling stability at the same reversible capacity of 1500 mAh/g for over 120 cycles with the low concentration of TTF (5 mM) (Fig. S9a), and for over 84 cycles at a low current density of 0.5 A/g (Fig. S9b), respectively, indicating the insignificant influences of the TTF concentration and the current density on the battery performances. Therefore, the highly enhanced reversible capacity and long cycling stability of the *h*-NPG based Li-O_2_ battery could be attributed to the unique and hierarchical nanoporosity structure of the NPG cathode. The large pores provide a fast path for oxygen and Li ion diffusion and a large open space for solid Li_2_O_2_ storage while the high effective surface area that mainly contributed by the smaller pores can promote the formation of thinner Li_2_O_2_ films for easy decomposition at low charge/discharge overpotentials. Moreover, the TTF kinetically profits the decomposition of Li_2_O_2_ within nanopores for a high capacity at lower charge overpotentials.

We investigated the rate dependence of the *h*-NPG based Li-O_2_ battery at varied current densities from 0.2 to 10.0 A/g with a cut-off capacity of 500 mAh/g (Fig. 3c). The discharge potential gradually decreases from ~2.80 to 2.50 V while the charge potentials increase from ~3.40 to 3.65 V as the current density increases from 0.2 to 10.0 A/g (Fig. 3d), showing a typical rate dependence of polarization resistance. It is worth noting that the charge potential is only ~3.65 V even at the high current density of 10.0 A/g, which is significantly lower than that of the carbon based electrodes which is usually above ~4.5 V at the similar current density. Additonally, the discharge potential keeps higher than those of the carbon electrodes, giving rise to a high energy efficiency of the *h*-NPG based Li-O_2_ battery. When the current density is set back to 1.0 A/g after the rate change tests, the discharge/charge potentials are identical to the initial ones at 1.0 A/g. This performance further confirms the excellent stability and feasibility of the *h*-NPG based Li-O_2_ battery. The low rate dependence of the discharge/charge potentials has also been verified with a large cut-off capacity of 1500 mAh/g at the current densities of 0.5 and 3.0 A/g (Fig. S9c). The energy efficiency of the Li-O_2_ battery at different current densities is plotted in Fig. 3d. With the current density increasing from 0.2 to 1.0 A/g, the energy efficiency of the *h*-NPG based Li-O_2_ battery gradually decreases from ~82% to 76%, which are much higher than those (~55–65%) of carbon-based Li-O_2_ batteries^3^,^5^.

**Figure 4:**
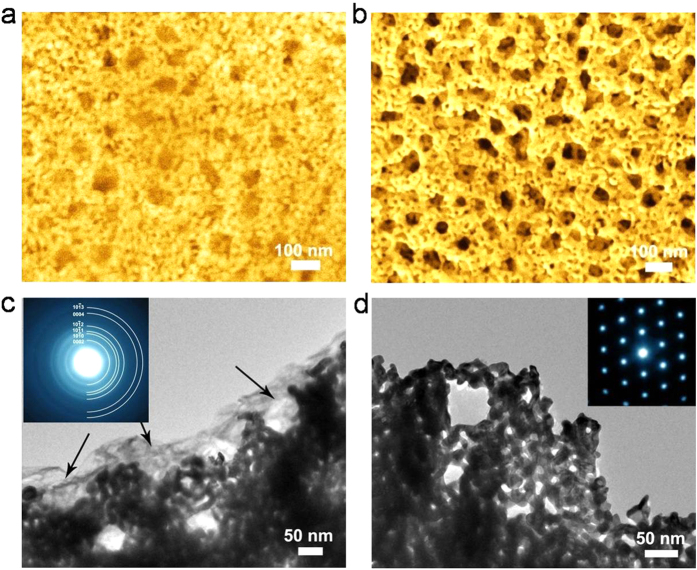
(**a**) SEM images of a discharged hierarchical NPG cathode. (**b**) SEM micrograph of a fully charged cathode. (**c**) Corresponding bright-field TEM image of the discharged hierarchical NPG cathode and the SAED pattern taken from the reaction products. (**d**) Bright-field TEM image and corresponding SAED pattern of a hierarchical NPG cathode after fully charging. The charged and discharged cathodes were prepared with the cut-off capacity of 1500 mAh/g at the current density of 0.5 A/g.

The *h*-NPG cathodes have been further inspected by the SEM and transmission electron microscopy (TEM) after the battery was cycled with the cut-off capacity of 1500 mAh/g. From the SEM of the discharged electrode (Fig. 4a), it can be observed that the solid reaction products uniformly grow in the porous electrode and the fuzzy contrast is probably caused by the insulator nature of the reaction products and extremely small Li_2_O_2_ grain sizes. After recharging (Fig. 4b), the fuzzy reaction products are almost removed and the SEM image of the electrode is the nearly same as the as-prepared *h*-NPG. The discharged and charged NPG electrodes were also inspected by TEM. As shown in Fig. 4c, the reaction products homogeneously distribute in the nanopores with a relatively bright contrast. The selected area electron diffraction shows the ring patterns of the reaction products, which can be index as the lithium peroxide (Li_2_O_2_) nanocrystals with a hexagonal (*P6*_*3*_*/mmc*) crystal structure (Inset in Fig. 4c). The smooth electron diffraction rings indicate that the Li_2_O_2_ crystals have a very small grain size and random orientations. Separated high-resolution TEM confirms that the Li_2_O_2_ products have a crystalline grain size smaller than 10 nm formed at a discharge current density of 0.5 A/g. After recharging, the reaction products are removed from the cathode, which cannot be recognized from the bright-field TEM image and electron diffraction pattern of the charged sample (Fig. 4d). The direct microscopic observations demonstrate that the nanoporous structure of gold is advantageous for the preferential formation and decomposition of Li_2_O_2_ at low overpotentials by forming nano-sized reaction products as thin films on the electrode surface. The full reversibility of the *h*-NPG based Li-O_2_ battery is tested by the electrochemical impedance spectroscopy (EIS) at discharge/charge states. As shown in Fig. S10a, the impedance of the battery increases significantly after the discharge because of the formation of insulated Li_2_O_2_ on the *h*-NPG cathode. After recharging, the impedance is fully recovered to the initial state, demonstrating that the Li_2_O_2_ is fully decomposed after recharging. Furthermore, XPS measurements confirm that the discharge product is pure Li_2_O_2_ and no other side reaction products (Fig. S10b). After recharging, Li_2_O_2_ can be completely decomposed, indicating the excellent reversibility of the *h*-NPG cathode.

The stability of TTF and the DMSO-based electrolyte have been explored by ^1^H NMR and GC-MS. The ^1^H NMR spectra of the TTF for the 1^st^, 50^th^ and 100^th^ cycles are obtained (Fig. S11) and show that there is no obvious change of the TTF peak after long cycles, indicating that the large reversible capacity with excellent cycling stability of the *h*-NPG based Li-O_2_ battery was not caused by the TTF oxidation/decomposition. Similarly, the stability of DMSO-based electrolyte has also been confirmed by the ^1^H NMR spectra (Fig. S12a). However, by the GC-MS with much higher sensitivity, a very low amount of byproducts (Dimethyl Sulfone) indicated by the weak peak can be observed after 100 cycles, demonstrating that the decomposition of DMSO in each cycle is very limited and accumulated to only ~2% after100 cycles (Fig. S12b). Both of the H NMR and GC-MS have revealed that the large reversible capacity with good cycling stability of the *h*-NPG based Li-O_2_ battery was contributed by the reversible Li_2_O_2_ formation/decomposition in the hierarchical nanoporosity of the cathode.

## Discussion

For conventional carbon based electrodes, they usually require binders, which makes the porosity tuning become very difficult in a controllable manner. In our study, it is the first report on the morphology tuning of NPG for the application in Li-air batteries. The free-standing NPG cathode with a uniform porous structure allows us to precisely control the pore size, morphology and porosity to uncover the intrinsic correlation of these porous parameters with batteries performances. More importantly, the hierarchical nanoporous structure developed in this study effectively overcomes the dilemma issue in specific surface area and porosity of conventional porous materials. Apparently, a large effective surface area benefits the reaction kinetics of Li_2_O_2_ formation and decomposition during discharging and charging while high porosity with the low mass density can host more Li_2_O_2_ for a high specific capacity. However, it is usually intolerable to realize both high porosity and large surface area simultaneously in a porous material because larger surface areas need a smaller pore size while higher porosity requires larger pores. The hierarchical porosity offers both high surface area and large porosity simultaneously which give rise to the excellent battery performances of the *h-*NPG cathode, in comparison with all previously reported nanoporous gold based Li-air batteries. Moreover, since the porous parameters of NPG can be well controlled by the dealloying method in comparison with porous carbon materials, dealloyed NPG is an excellent model system to explore the intrinsic correlation between porous structure and battery performance. In this sense, the current results from a simple and clean system is extremely valuable and could be as a universal law to guide the future cathode design regardless of materials.

From practical application viewpoint, gold appears expensive and it seems not practical to use NPG in large-scale commercial Li-O_2_ batteries. However, this may not be true for *h*-NPG. After introducing up to 80% porosity by the hierarchical configuration, the loading amount of gold in a cathode becomes very low. In term of the efficiency of the cathode materials, the material cost of *h*-NPG is actually obviously lower than those of the carbon-RuO_2_ cathode, the most promising one for practical applications in Li-O_2_ batteries (Table S3). Therefore, the hierarchical configuration not only provides excellent battery performances, but also benefits the material costs of cathodes by increasing porosity and decreasing loading amount of cathode materials.

In summary, we have developed a hierarchical NPG with a large surface area and high porosity for Li-O_2_ battery. A highly enhanced reversible capacity and long cycling lifetime can be simultaneously achieved at low charge/discharge overpotentials and a high current density with the assistance of the redox mediator TTF. The *h*-NPG based Li-O_2_ battery also shows much high energy efficiency and extraordinarily low rate dependence. Since the *h*-NPG electrode used in the battery is free-standing, it can work as the current collectors simultaneously in addition to the high-performance cathodes. The excellent battery performance of this model system demonstrates that the capacity and stability of Li-O_2_ batteries can be dramatically improved by enlarging both porosity and specific surface area with the hierarchical porous structure. The large porosity introduced and stabilized by the hierarchical configuration also significantly increases the efficiency of cathode materials and thus reduces the material costs by decreasing the loading amount of cathode materials. Therefore, this study may pave a new way to develop high capacity and highly stable nanoporous metals with low materials costs as cathodes for full-performance metal-O_2_ (Li-O_2_ and Na-O_2_) batteries. Moreover, the realization of enhanced battery properties by optimizing the nanoporous morphology has an important implication in developing high performance cathodes by designing hierarchical porous structure.

## Methods

### Materials

Commercial Au_15_Ag_85_ (at%) alloy leaves with a thickness <1 μm were used as precursors to fabricate hierarchical NPG by a two-step dealloying process^49^. The Au_15_Ag_85_ alloy was first electrochemically dealloyed in a 1M HNO_3_ solution at 0.78 V for 900s using an electrochemical workstation (Ivium Technology) in a standard three-electrode cell with a Ag-AgCl reference electrode and a Pt counter electrode. The dealloyed samples (as-prepared np-AuAg) were rinsed by deionized water (18.2 MΩ·cm) for more than three times to remove the residual chemical substances in the pore channels. Subsequently, the as-prepared np-AuAg films were annealed at the temperature of 250 °C for 1 h. The annealed np-AuAg films with coarsened nanopores and ligaments were further electrochemically dealloyed at a higher potential of 0.98 V for 1200s. By the two-step delloying, a hierarchical nanoporous structure with large primary pores of ~80–100 nm and small secondary pores of ~5–20 nm can be fabricated. The surface areas of the as-prepared np-AuAg, coarsened np-AuAg and hierarchical NPG were measured to be ~59.8, 27.4 and 82.9 m/g in 0.5M H_2_SO_4_ in the potential range of 0.0–1.5 V_Ag/AgCl_ at the scan rate of 100 mV/s, respectively (Fig. S2b). The mass of the electrodes (~0.2–1.0 mg) was measured with an ultra-micro balance. The geometric surface areas were ~1.0 cm^2^. After dealloying the thickness of the samples has almost not changed. NPG films with the nearly same Ag concentration as the hierarchical NPG were prepared by one-step electrochemical dealloying of Au_15_Ag_85_ alloy at 0.82 V *vs.* Ag/AgCl for 1.5 h at room temperature. The as-prepared NPG was then annealed at 200 °C to obtain the coarsened nanoporous structure.

### Microstructural characterization

The microstructures of the NPG cathodes and reaction products were investigated by a field-emission scanning electron microscope (JEOL JIB-4600F SEM, 15 keV) equipped with an energy dispersive X-ray spectrometer and a transmission electron microscope (JEOL JEM-2100F) with double spherical aberration correctors for both the probe-forming and image forming objective lenses. The discharge products were characterized at a low operation voltage of 120 keV to avoid the electron beam damage.

### Electrochemical characterization

The R2032-type coin batteries (MTI Corporation) were assembled using the as-prepared np-AuAg, coarsened np-AuAg and hierarchical NPG films as the working electrodes and pretreated Li metal plates as the anodes^47^. Distilled DMSO was used as the solvent to make 1 M LiClO_4_ electrolyte with and without the redox mediator TTF^48^. The cells were assembled in an Ar-filled glove box (water content <1 ppm) by stacking a Li metal anode, a Whatman glass fiber separator and a NPG cathode. The assembled Li-O_2_ battery was tested at room temperature in a glass chamber with ~1.0 atm O_2_ (>99.99995%) flowing. Galvanostatic discharge/charge tests were conducted by using a Hokuto discharging/charging system. The current density and specific capacity were both calculated based on the loading mass of NPG or np-AuAg. Discharged and recharged NPG cathodes were inspected by SEM and TEM after the electrolyte was washed away in a glove box.

Cyclic voltammetry (CV) measurements were carried out in a non-aqueous 1.0 M LiClO_4_/DMSO electrolyte using a three-electrode electrochemical system (Fig. S7)^22^. Hierarchical NPG films were mounted on a glassy carbon disk electrode (5 mm in diameter) by lithiated Nafion^®^ (LITHion dispersion, Ion-Power, USA). A freshly polished lithium foil was employed as the counter electrode. The reference electrode was based on a silver wire immersed in 0.1M TBAPF_6_ (Sigma-Aldrich) and 0.01 M AgNO_3_ (BASi) in DMSO which was calibrated against Li metal in 1M LiClO_4_/DMSO (0 V_Li_ = ~−3.38 ± 0.01 V *vs.* Ag/Ag^+^). The electrolyte was bubbled with pure O_2_ for 30 min before CV measurements. To test the TTF effect, 50 mM TTF was added into the 1.0 M LiClO_4_/DMSO electrolyte.

## Additional Information

**How to cite this article**: Guo, X. W. *et al*. Hierarchical nanoporosity enhanced reversible capacity of bicontinuous nanoporous metal based Li-O_2_ battery. *Sci. Rep.* **6**, 33466; doi: 10.1038/srep33466 (2016).

## Supplementary Material

Supplementary Information
