# Supplementary material for: Hierarchical nanoporosity enhanced reversible capacity of bicontinuous nanoporous metal based Li-O2 battery
Source: Sci Rep. 2016 Sep 19;6:33466. doi: 10.1038/srep33466 (PMC5027567; doi:10.1038/srep33466)
Supplement: Supplementary Information [file srep33466-s1.pdf]

# Supplementary Information

# Hierarchical nanoporosity enhanced reversible capacity of bicontinuous nanoporous metal based Li-$O_2$ battery

Xianwei Guo[1,2†], Jiuhui Han[1,†], Pan Liu[1,2†], Luyang Chen[1], Yoshikazu Ito[1,2], Zelang Jian[3], Tienan Jin[1], Akihiko Hirata[1,2], Fujun Li[3], Takeshi Fujita[1], Naoki Asao[1], Haoshen Zhou[3], Mingwei Chen[1,2,4]*

1 WPI Advanced Institute for Materials Research, Tohoku University, Sendai 980-8577, Japan

2 CREST, JST, 4-1-8 Honcho Kawaguchi, Saitama 332-0012, Japan

3 Energy Technology Research Institute, National Institute of Advanced Industrial Science and Technology (AIST), Umezono 1-1-1, Tsukuba, 305-8568, Japan

4 School of Materials Science and Engineering, Shanghai Jiao Tong University, Shanghai 200030, PR China

[†] These authors contributed equally to this work.

*E-mail: mwchen@wpi-aimr.tohoku.ac.jp

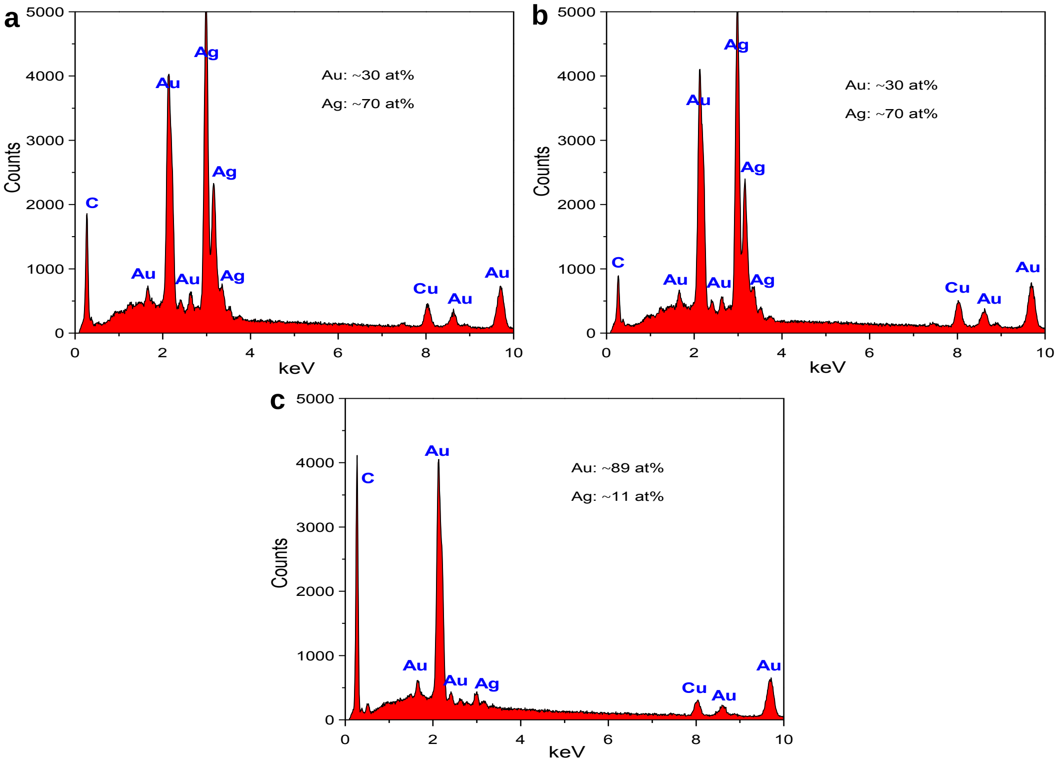


**Fig. S1** Energy dispersive X-ray spectroscopy (EDS) spectra of (**a**) as-prepared np-AuAg; (**b**) coarsened np-AuAg; and (**c**) hierarchical NPG. The Cu peaks are from the copper sample holder.

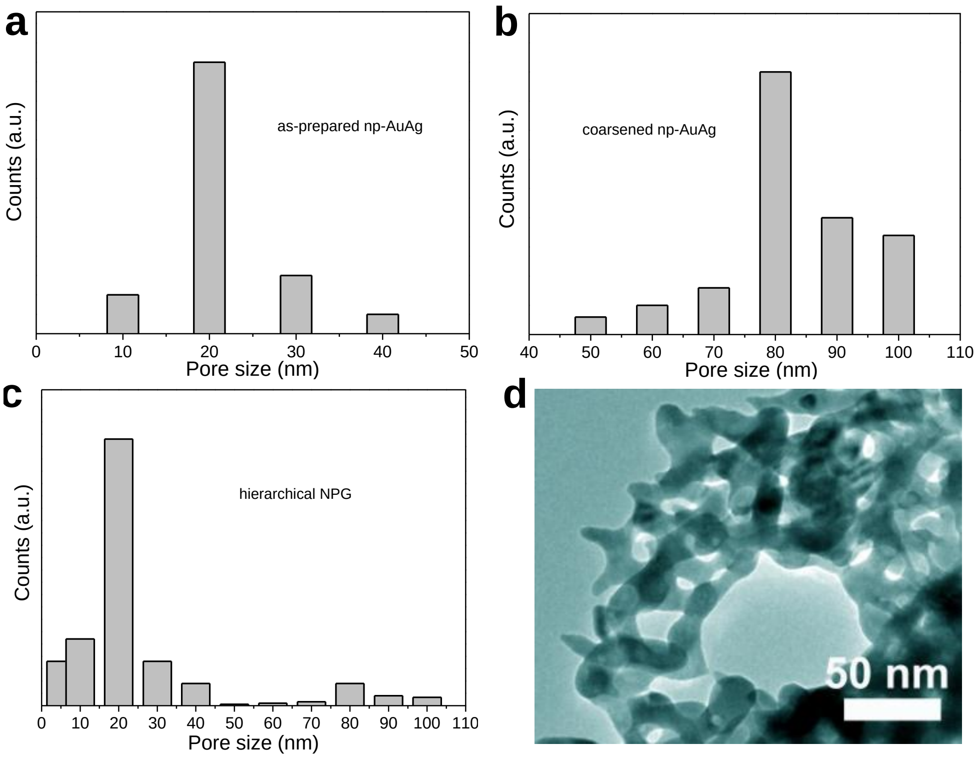


**Fig. S2** The pore size distributions of three different electrodes (**a**) as-prepared np-AuaG, (**b**) coarsened np-AuAg, (**c**) hierarchcial NPG. (**d**)**,** The TEM image of the hierarchical nanoporous gold.

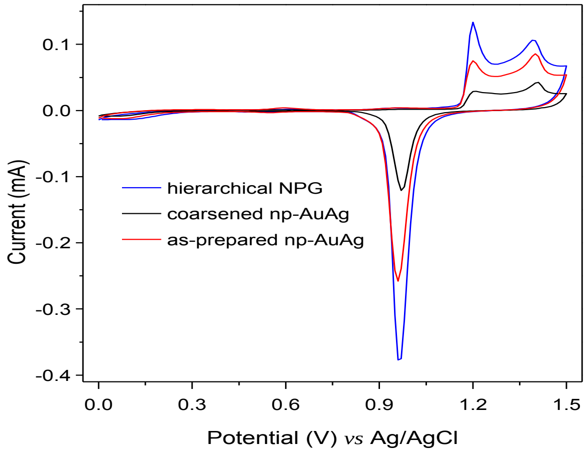


**Fig. S3** Cyclic voltammetric curves of as-prepared and coarsened np-AuAg as well as hierarchical NPG, recorded at the potential ranging from 0.0 to 1.5 $V_{Ag/AgCl}$ at the scan rate of 100 mV/s in Ar-saturated 0.5 M $H_2SO_4$. The electrochemically effective surface areas of the three samples were determined by integrating the net charge formation of surface oxides.

**Table S1** Chemical compositions and geometric parameters of the three nanoporous electrodes.

| | Composition (at%) (±2 at%) | Porosity (vol.%) | Pore size (nm) | Electrochemical surface area ($m^2$ $g^{-1}$) |
|---|---|---|---|---|
| as-prepared np-AuAg | $Au_{30}Ag_{70}$ | ~50 | ~20 | ~59.8 |
| Coarsened np-AuAg | $Au_{30}Ag_{70}$ | ~50 | ~80-100 | ~27.4 |
| hierarchical NPG | $Au_{89}Ag_{11}$ | ~80 | ~5-20<br>~80-100 | ~82.9 |

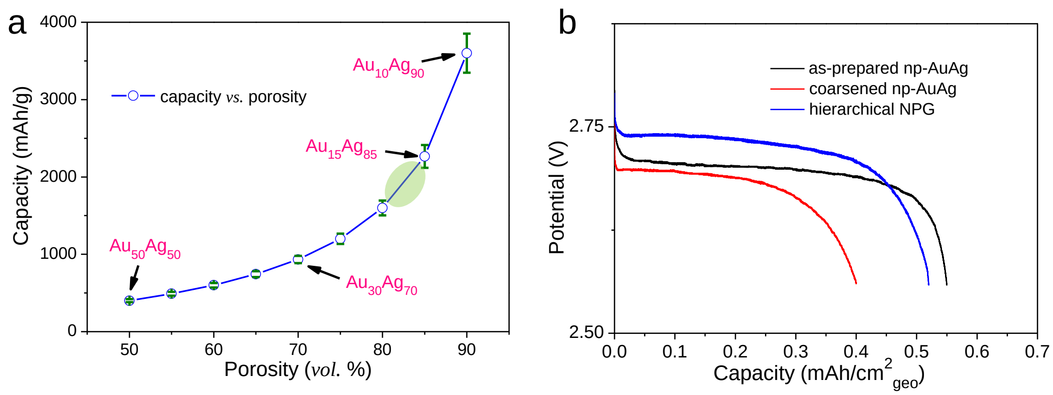


**Fig. S4** (a) The relationship between the discharge capacity and the porosity by the theoretical calculations in the NPG based Li-$O_2$ battery. From the Sciences (373 (2012) 563-566) report, the full discharge capacity of $Au_{50}Ag_{50}$ is about 350 mAh/g. The other capacities are calculated by the formula: 350P/(1-P), where P is the theoretical porosity ratio after removing all the Ag in the alloys. (b) The maximum capacities normalized by the geometric surface areas of the three NPG cathodes based on **Figs.2b-c**.

**Table S2** The measured overpotentials of the three np-AuAg and NPG electrodes based on **Fig. 2**. The basic electrolyte is 1M $LiClO_4$ in DMSO. The values are taken from the difference of the terminal or middle potentials (charge or discharge) from the thermodynamic potential ($U_0$= 2.96V).

| | Overpotentials without TTF (V) | | | | Overpotentials with TTF (V) | | | |
|---|---|---|---|---|---|---|---|---|
| | Terminal (±0.01V) | | Middle (±0.01V) | | Terminal (±0.01V) | | Middle (±0.01V) | |
| | $\eta_{discharge}$ | $\eta_{charge}$ | $\eta_{discharge}$ | $\eta_{charge}$ | $\eta_{discharge}$ | $\eta_{charge}$ | $\eta_{discharge}$ | $\eta_{charge}$ |
| as-prepared np-AuAg | 0.25 | 0.90 | 0.24 | 0.83 | 0.26 | 0.56 | 0.25 | 0.52 |
| coarsened np-AuAg | 0.28 | 0.97 | 0.27 | 0.88 | 0.28 | 0.60 | 0.27 | 0.57 |
| hierarchical NPG | 0.22 | 0.83 | 0.21 | 0.71 | 0.23 | 0.51 | 0.23 | 0.47 |

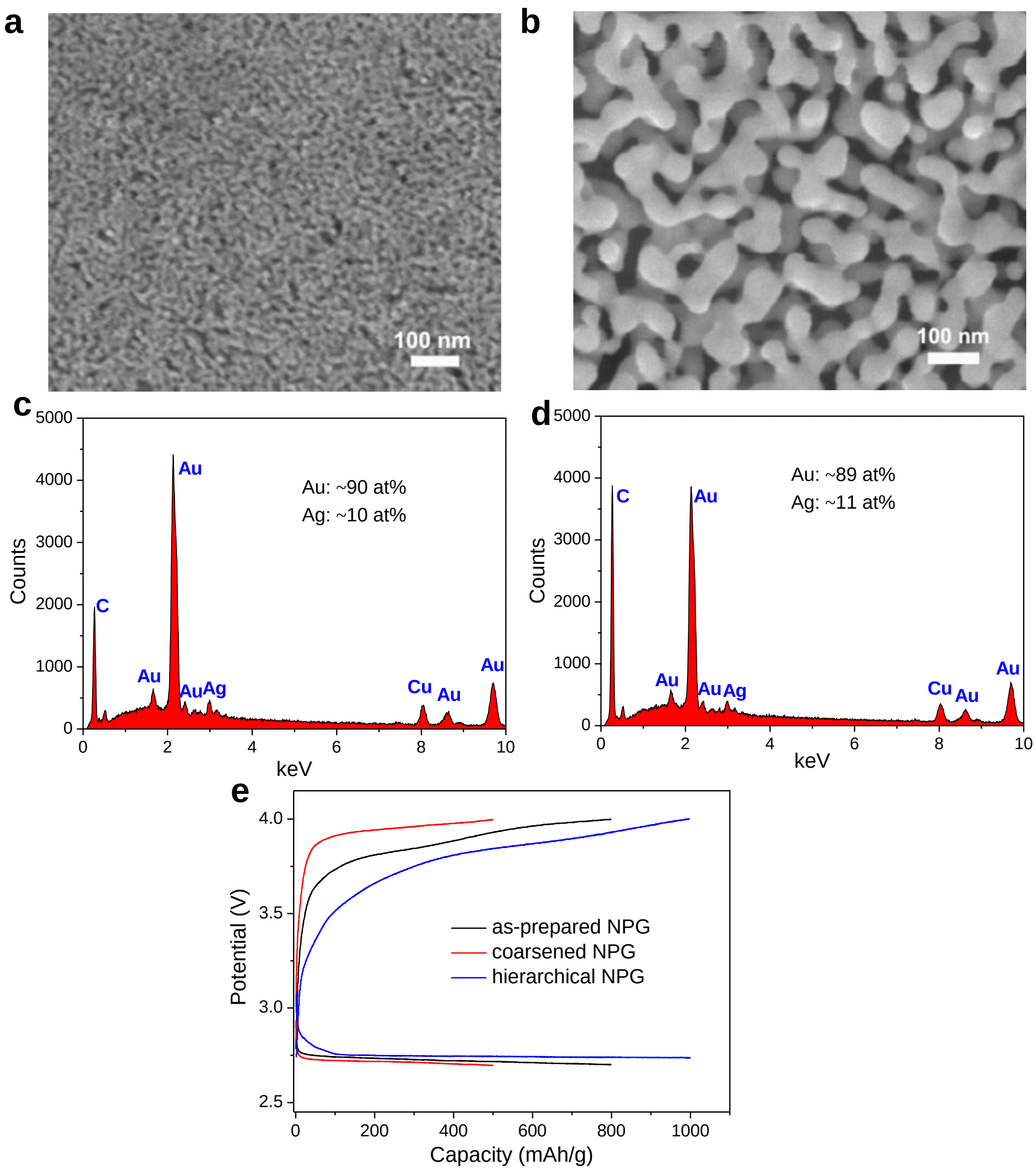


**Fig. S5** The corresponding SEM images of the (**a**) as-prepared and (**b**) coarsened NPG prepared by one-step electrochemical dealloying. The samples have almost identical nanopore sizes as those of the as-prepared and coarsened np-AuAg. Energy dispersive X-ray spectroscopy (EDS) spectra of the (**c**) as-prepared; and (**d**) coarsened NPG. The residual Ag concentration (~10 at%) is nearly the same as the hierarchical NPG. The Cu peaks are from the copper sample holder. **e,** The stable discharge/charge curves of the as-prepared, coarsened and hierarchical NPG cathodes under the conditions (in 1 M $LiClO_4$/DMSO electrolyte at the current density of 0.5 A/g) identical to those of **Fig. 2d** in the main text. It is worth noting that the hierarchical NPG still shows the highest

activities for both charging and discharging reactions, followed by as-prepared NPG with small pores and then coarsened NPG. Since the three cathodes have almost the identical chemical composition, the electrode performance difference is mainly from the variation of nanoporous structure.

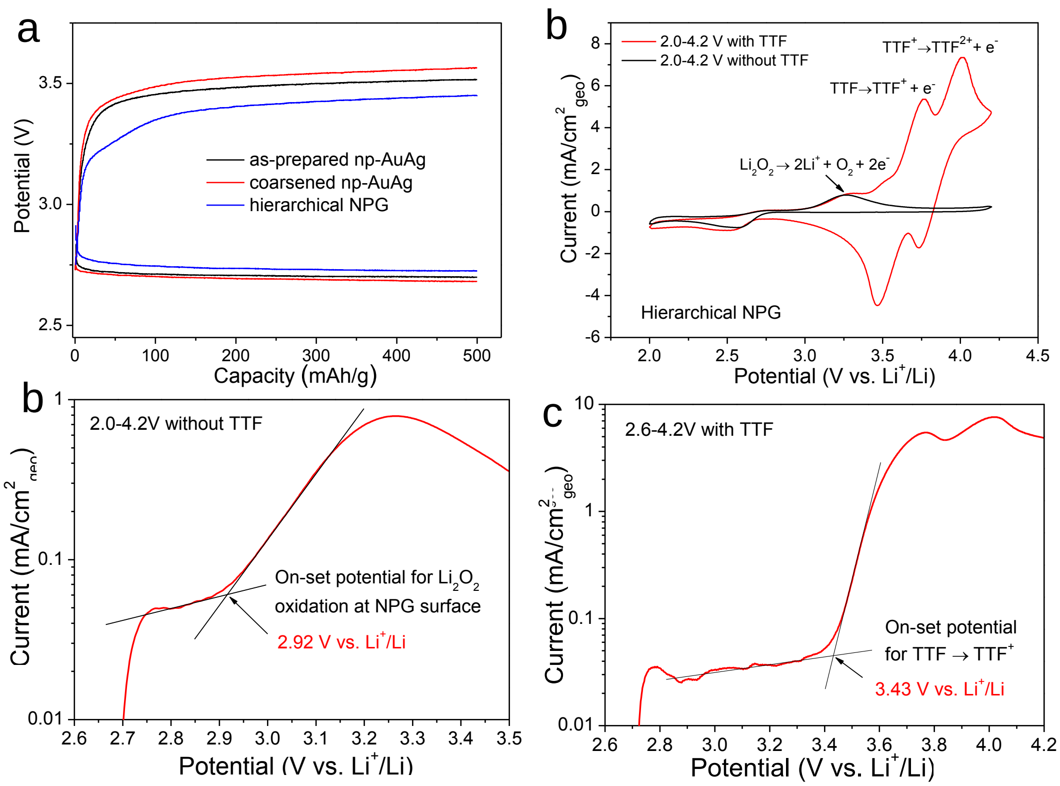


**Fig. S6** (**a**), The discharge/charge curves with the cut-off capacity of 500 mAh/g in 1M $LiClO_4$/DMSO electrolyte containing 50 mM TTF at the current density of 0.5 A/g. (**b**), Cyclic voltammetry (CV) profiles of a hierarchical NPG electrode collected at 100 mV/s in an oxygen-saturated 1M $LiClO_4$/DMSO electrolyte with 50 mM TTF at different potential ranges (with a constant upper potential limit of 4.2 V *vs.* $Li^+$/Li). The CV profile collected in the electrolyte without TTF was shown for comparison. (**c**), Onset potentials of the $Li_2O_2$ oxidation on hierarchical NPG cathode without TTF. (**d**), Onset potential of TTF oxidation. Note that the curve collected at the potential range 2.6-4.2 V was used for analyzing the onset potential for TTF oxidation because almost no $Li_2O_2$ forms on the electrode at this potentials range.

**Fig. S6b** shows the CV profiles collected at the scan potential window of 2.0-4.2 V. In addition to the four apparent peaks corresponding to the redox couples of TTF/$TTF^+$ and $TTF^+$/$TTF^{2+}$ at the high potential region of the CV profiles, several peaks, intrinsically different from those of TTF redox couples, at ~2.5 V and ~3.2-3.4 V can also be

observed at the low potential region, which are associate with the oxygen reduction ($Li_2O_2$ formation) and $Li_2O_2$ oxidation reactions, respectively. The oxygen reduction processes and peak potentials (~3.2-3.4 V) for $Li_2O_2$ oxidation on NPG do not show obvious difference in electrolytes with and without TTF except a slight shift in the current density of the cathiodic sweep, and the CV curves are completely overlapped from the onset potentials to the peak potentials of the $Li_2O_2$ oxidation (**Fig. S6b**), suggesting that the oxidation reaction at the potentials below ~3.3 V (the peak potential of $Li_2O_2$ oxidation) is mainly governed by the operation of $Li_2O_2/O_2$ couples on NPG while TTF does not play a noticeable role. This is in accordance with the measured onset potentials of ~2.9 V for $Li_2O_2$ oxidation on *h*-NPG surfaces and ~3.4 V for TTF oxidation, respectively (**Figs. S6c-d**). The effect of TTF upon the oxidation of $Li_2O_2$ is most likely associated with a shoulder peak at ~3.5 V right after the direct $Li_2O_2$ oxidation peak (**Fig. S6b**). It only shows up together with the redox peaks of $TTF/TTF^+$ and $TTF^+/TTF^{2+}$ and cannot be seen in the CV curves tested from the electrolyte without TTF. Thus, the TTF ($TTF^+$) enhanced $Li_2O_2$ oxidation takes place at the potentials above ~3.4 V, which contributes to the majority of the charge capacity achieved by the *h*-NPG based Li-$O_2$ battery as shown in **Fig. 3a**.

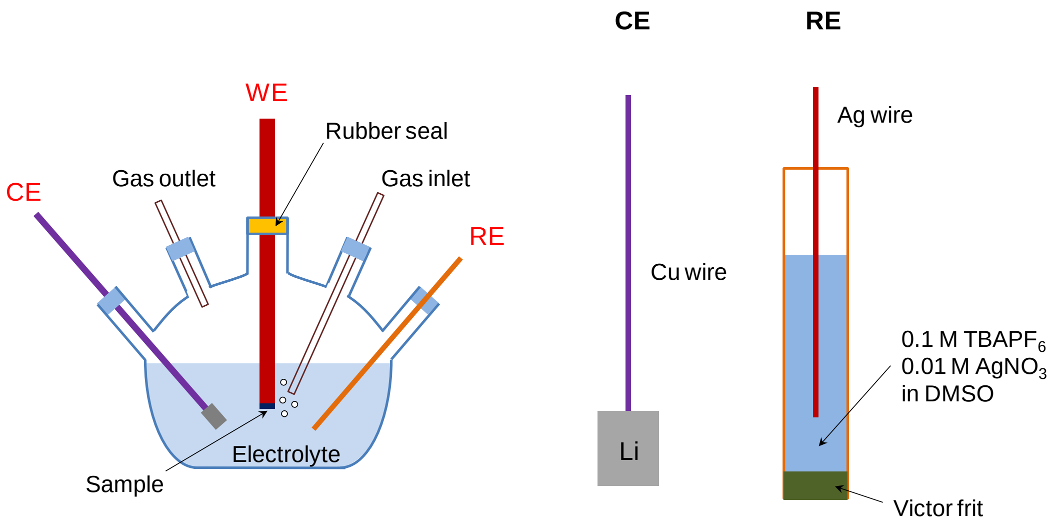


**Fig. S7** Schematic diagram of the home-built three-electrode electrochemical system for the *cyclic voltammetry* measurements. The cell consists of a freshly polished lithium-foil counter electrode, a reference electrode based on a silver wire immersed in 0.1M $TBAPF_6$ (Sigma-Aldrich) and 0.01M $AgNO_3$ (BASi) in DMSO which was calibrated against Li metal in 1M $LiClO_4$/DMSO (0 $V_{Li}$ = ~ -3.38 ± 0.01 V *vs.* $Ag/Ag^+$), and a thin-film working electrode. The working electrode was prepared by mounting the NPG film onto a glassy carbon disk electrode (5 mm in diameter) by lithiated Nafion® (LITHion™ dispersion, Ion-Power, USA). The three-electrode system is airtight and assembled and sealed inside an Ar-filled glove box. Gas inlets/outlets were designed which allows the bubbling of electrolyte with Ar and $O_2$ gases.

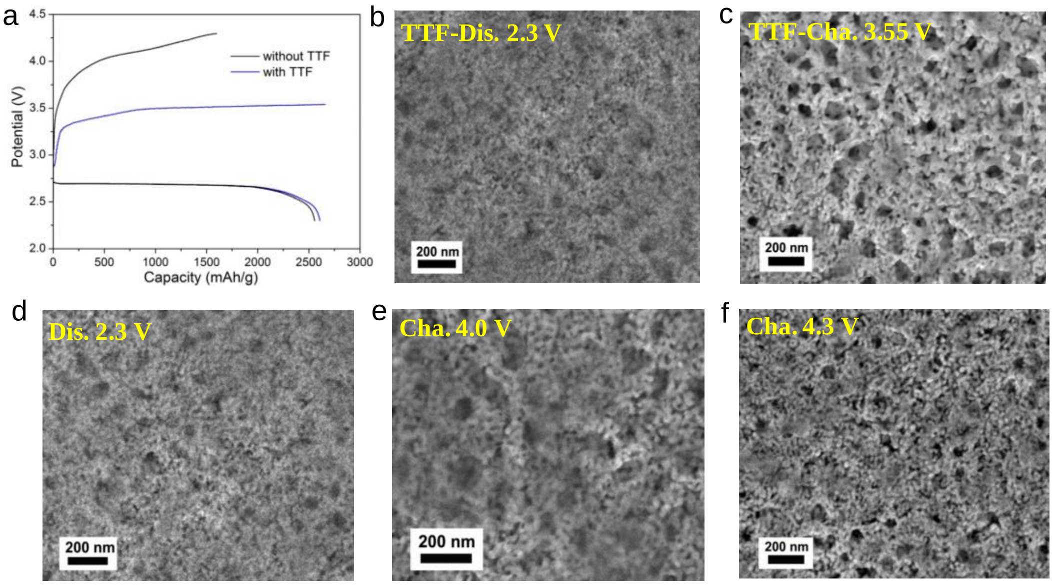


**Fig. S8** (**a**), The full discharge/charge curves of the hierarchical NPG-based Li-$O_2$ battery in 1M $LiClO_4$/DMSO electrolyte with or without 50 mM TTF at the current densities of 0.5 A/g. The SEM images of the hierarchical NPG cathode at the (**b**), fully discharged and (**c**), fully recharged states with the TTF. The SEM images of the hierarchical NPG cathode at the (**d**), fully discharged and (**e-f**), recharged to different potentials without the TTF.

As shown in **Fig. S7a**, the battery shows the fully reversible capacity at the charge potential of 3.55 V with the TTF. However, without the TTF, the battery can only reach ~1500 mAh/g at the high cut-off charge potential of 4.3 V, indicating that the TTF can be effective in enhancing the decomposition of $Li_2O_2$ at low charge potential, and no influence on the discharge with the maximum capacity. The solid $Li_2O_2$ on the *h*-NPG cathodes are also observed directly by the scanning electron microscopy (SEM). From the SEM of the discharged NPG electrode (**Fig. S7b**), the solid reaction products grow on the porous electrode and the pores are fully filled by insulating $Li_2O_2$. After the battery was charged to 3.55 V with the TTF (**Fig. S7c**), the reaction products are almost removed. However, if there is no TTF in the electrolyte, there are many $Li_2O_2$ residuals in the nanopores even after the battery was charged to 4.3 V (**Figs. S7d-f**), consistent with the discharge/charge curves in **Fig. S7a**.

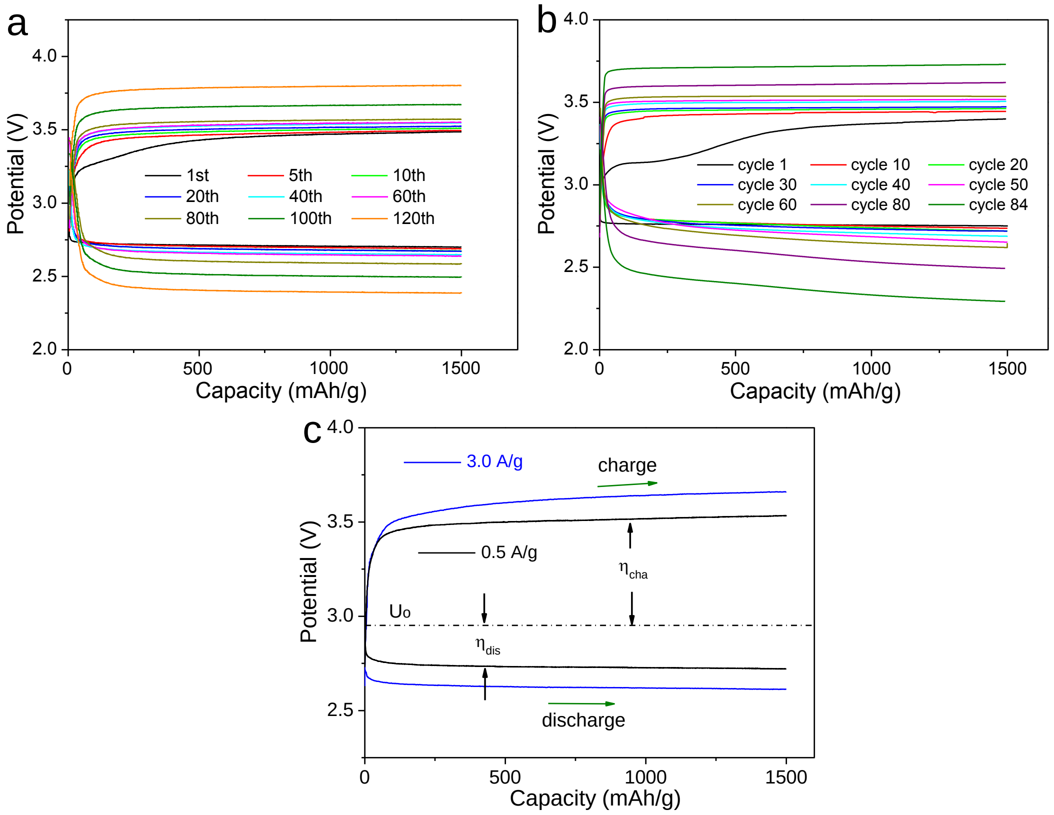


**Fig. S9 (a)**, The galvanostatic discharge/charge curves of the hierarchical NPG cathode at the current density of 2.0 A/g with the cut-off capacity of 1500 mAh/g. The electrolyte is 1 M $LiClO_4$ in DMSO with 5 mM TTF. (**b**), The galvanostatic discharge/charge curves of the hierarchical NPG cathode at the current density of 0.5 A/g with the cut-off capacity of 1500 mAh/g. The electrolyte is 1 M $LiClO_4$ in DMSO with 50 mM TTF. (**c**), The galvanostatic discharge/charge curves of the hierarchical NPG-based Li-$O_2$ battery with the cut-off capacity of 1500 mAh/g at the current densities of 0.5 and 3.0 A/g; and the definition of the discharge/charge overpotentials in a galvanostatic curve.

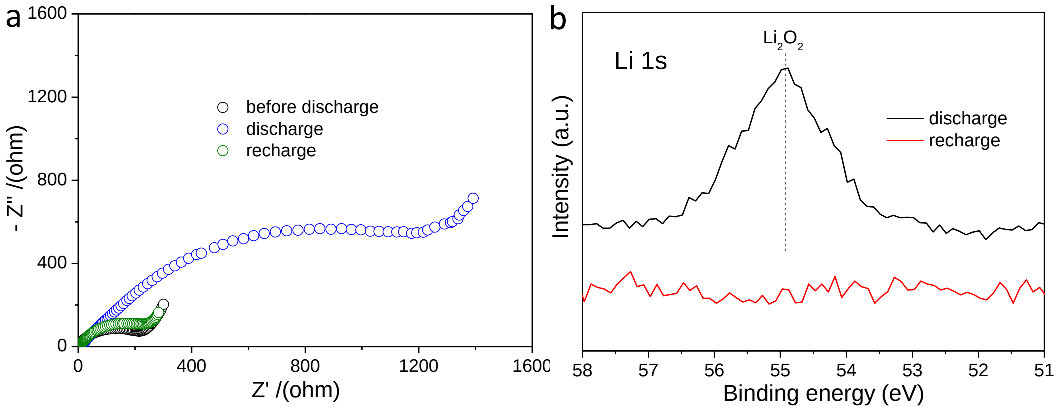


**Fig. S10** (a) Electrochemical impedance spectra of *h*-NPG based Li-$O_2$ battery before test and at 1$^{st}$ discharge/charge stages with the capacity of 1500 mAh/g at the current density of 500 mA/g. (b) Li 1s XPS spectra of the h-NPG cathode at the discharge and charge states with the cutoff capacity of 1500 mAh/g at the current density of 500 mA/g.

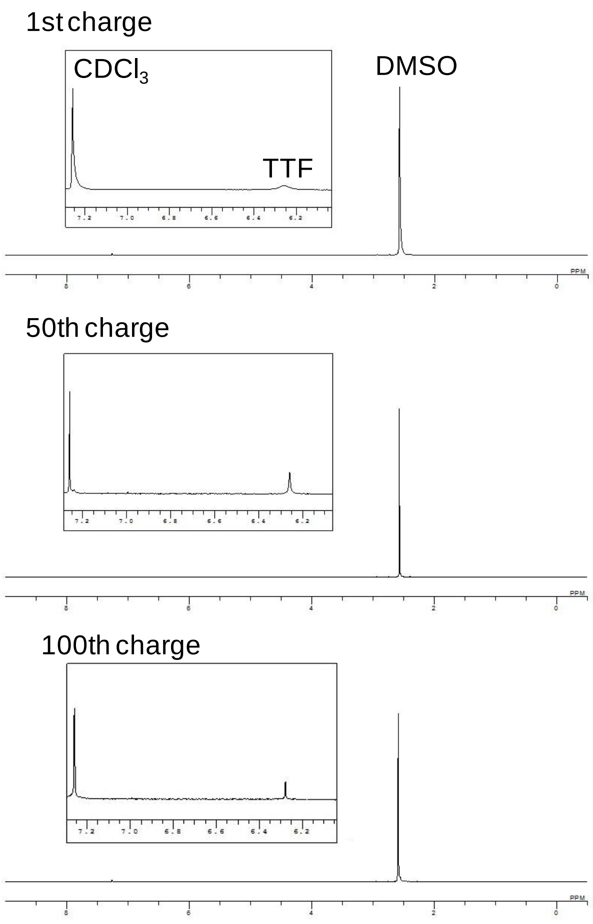


**Fig. S11** $^1$H NMR of the TTF redox mediator in the hierarchical NPG based Li-$O_2$ battery at the cut-off capacity of 1500 mAh/g after different cycles. The DMSO was shown for conference.

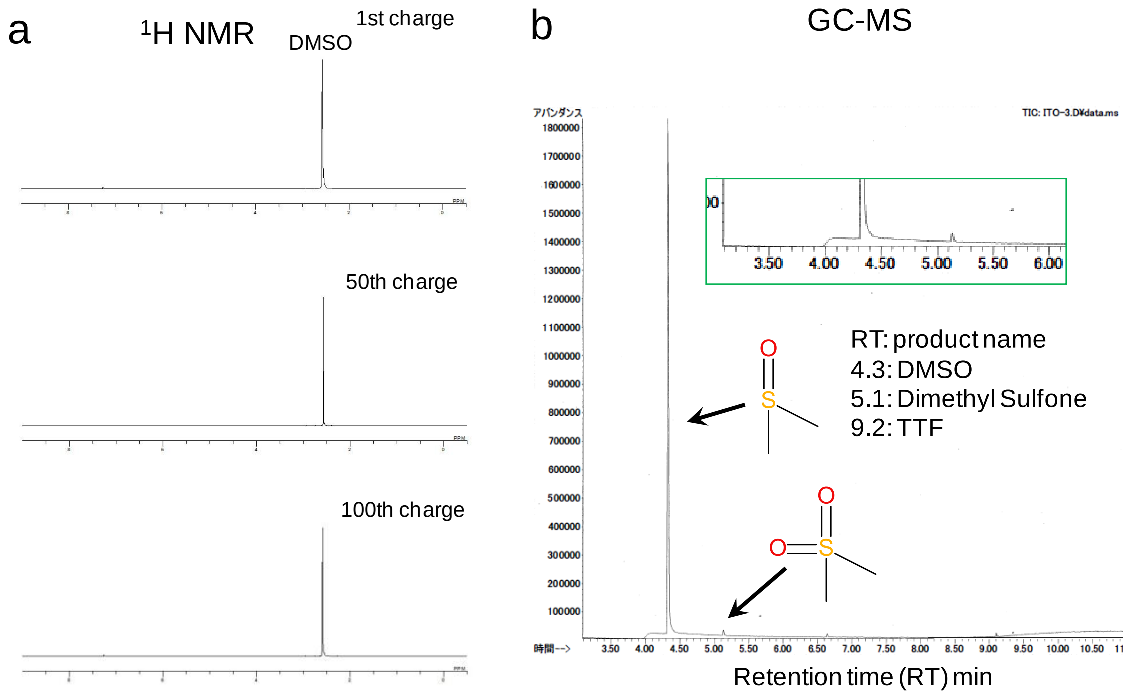


**Fig. S12 (a)**, $^{1}$H NMR of the stability of DMSO in the hierarchical NPG based Li-$O_2$ battery at the cut-off capacity of 1500 mAh/g after different cycles. (**b**), The byproduct of DMSO decomposition after 100 cycles was revealed by GC-MS.

**Table S3** The materials costs of different cathodes with catalysts. The efficiency of the cathode materials was calculated by the price of the loading $RuO_2$ per gram with a maximum discharge capacity.

| Cathodes | Maximum discharge capacity (MDC) | Price of loading catalyst ($/g) | Price/MDC ($) |
|---|---|---|---|
| Hierarchicl NPG | 2400 mAh/$g_{total}$ | 42.86 | 0.017 |
| CNT-74wt% $RuO_2$ [26] | 1200 mAh/$g_{total}$ | 81.30 | 0.068 |
| CNT-32wt% $RuO_2$ [R1] | 1200 mAh/$g_{total}$ | 35.15 | 0.03 |
| Graphene-40wt% $RuO_2$ [28] | 8700 mAh/$g_{total}$ | 43.94 | 0.005 |

**References:**

[R1] E. Yilmaz, C.Yogi, K.Yamanaka, T.Ohta, H. R. Byon, *Nano Lett.*, **2013**, *13*, 4679.
